# Privacy and Ethics in Pediatric Environmental Health Research—Part I: Genetic and Prenatal Testing

**DOI:** 10.1289/ehp.9003

**Published:** 2006-08-14

**Authors:** Celia B. Fisher

**Affiliations:** Center for Ethics Education, Fordham University, Bronx, New York, USA

**Keywords:** adolescents, confidentiality, environmental health, ethics, genetic determinants, informed consent, pediatric research, prenatal testing, privacy

## Abstract

The pressing need for empirically informed public policies aimed at understanding and promoting children’s health has challenged environmental scientists to modify traditional research paradigms and reevaluate their roles and obligations toward research participants. Methodologic approaches to children’s environmental health research raise ethical challenges for which federal regulations may provide insufficient guidance. In this article I begin with a general discussion of privacy concerns and informed consent within pediatric environmental health research contexts. I then turn to specific ethical challenges associated with research on genetic determinants of environmental risk, prenatal studies and maternal privacy, and data causing inflicted insight or affecting the informational rights of third parties.

At the dawn of the 21st century, increased political and media attention on existing and emerging ecologic hazards and environmental toxicants has rekindled public anxiety about the health consequences for future generations. These concerns have propelled research on genetic susceptibility and environmental conditions threatening the nation’s youth. Research approaches to confirmed, suspected, or as yet unidentified relationships between the environment and developmental disorders have included assessments of biologic mechanisms through which environmental toxicants affect children’s health, identification of populations that may be genetically susceptible to environmental diseases, and evaluation of interventions designed to mitigate harms associated with environmental hazards (Sharp 2003).

The pressing need for empirically informed public policies aimed at understanding and promoting children’s health has challenged environmental scientists to modify traditional research paradigms and reevaluate their roles and obligations toward research participants. New methodologic approaches to children’s environmental health research raise ethical challenges for which current federal regulations and organizational standards may not provide sufficient guidance. One such challenge is how to construct a cohesive ethical framework for protecting the privacy rights of children and families participating in the diverse set of methodologies, age groups, and populations characterizing pediatric environmental health research.

Data from environmental research can lead to policies preventing or remediating environmentally based developmental disorders. At the same time, disclosure of such information can lead to personal or group stigmatization, restriction in employment opportunities, or higher rates of or inability to obtain health insurance in both childhood and adulthood. Ethical concerns regarding the collection and risks of disclosure of private environmental health information not only revolve around the principle of individual autonomy but also relate to principles of beneficence (do good) and nonmaleficence (do no harm) (National Commission for the Protection of Human Subjects of Biomedical and Behavioral Research 1978). Thus, risks to participant privacy rights need to be considered in any risk–benefit justification for the conduct of research. Ethical challenges are compounded when poor and less powerful populations are recruited for environmental hazards research. Attention to genetic susceptibility and cultural practices associated with environmental disease in underserved groups can unintentionally promote existing health disparities by placing responsibility on the population rather than environmental policies.

The direct and indirect implications of privacy violations on the health and social welfare of children places the adequacy of privacy protections at the forefront of ethical concern in the design, implementation, and dissemination of research on children’s environmental diseases. In this article I begin with a general discussion of privacy concerns and informed consent within a pediatric environmental health research context. I then address specific ethical challenges associated with research on genetic determinants of environmental risk, prenatal studies and maternal privacy, and data causing inflicted insight or affecting the informational rights of third parties.

## What Is Private Information in Environmental Health Research?

Privacy in research refers to the right of an individual to make decisions concerning how much information about their physical status, health, social network, and thoughts and feelings will be shared with investigators. Environmental research involving children has the potential to collect biologic or behavioral information that is not otherwise publicly accessible or observable and to which patients or family members do not wish others, including investigators, to have access. Private information collected on health status, genetic makeup, and the social and physical environments in which children develop requires more than conventional privacy protections. First, diseases stemming from early exposure to environmental toxicants may emerge at various points of the lifespan. For this reason, pre- and postnatal data must be stored and reanalyzed across many years. Long-term stored data may produce information not originally anticipated by the investigators, and therefore not consented to by child participants or their guardians. Blanket permission for future analyses of such data may undermine participant privacy if information is not deidentified and the implications of future confidentiality risks cannot be adequately determined.

Second, when research involves adult participants, protection of privacy rights can usually be achieved through adequate informed consent procedures. However, informed consent does not provide the same protection for children. First, children < 18 years of age do not typically have the statutory legal right to consent. As a result, federal regulations [Department of Health and Human Services (DHHS) 2005; U.S. Environmental Protection Agency (EPA) 2006] require parental permission and in some cases child assent. Second, because of their developmental cognitive limitations, the informed assent of infants and very young children is not required or typically sought. Third, when assent is obtained from older children and adolescents, it is questionable whether they have the cognitive maturity and experience to understand the current and future implications of sharing private information. Although parent permission is a critical means of protecting the rights of all child participants, it is unlikely that parents can always anticipate how their children will feel about third-party knowledge of their private information as the children mature. Fourth, the genetic privacy of biologic relatives is also at risk when children or siblings are the source of genetic information because probabilistic inferences can be made about the genetic traits of family members. Similarly, epidemiologic survey questions may pose family risks when the questions ask children to describe items in their home or parental health-related behaviors [Institute of Medicine (IOM) 2005].

### Federal regulations

Under current federal regulations, an individual may be considered a human subject if identifiable private information is collected (DHHS 2005; U.S. EPA 2006). Under the Common Rule, “private information” includes information about behavior that occurs in a context in which an individual can reasonably expect that no observation or recording is taking place, and information that has been provided for specific purposes by an individual and that the individual can reasonably expect will not be made public (e.g., a medical record) [DHHS 2005, 45CFR46.102(f); U.S. EPA 2006, 40 CFR Part 9]. Defining what is private in environmental health research poses challenges under this regulation. For example, if parents smoke only in the house, but not at work or in public places, are their smoking habits a private act? If farming families track pesticide-contaminated mud into their home and do not clean the floors on which their children play, is this a private act? Does it depend on whether the behaviors were or can be observed by family members or neighbors?

The scientific community has not reached consensus on distinctions between the public and private health-related behaviors of family members. Some have argued that health information is not private if known by family members (American Society of Human Genetics 2000). Others have argued that disclosures to one’s intimates should not be considered the same as public disclosures (Botkin et al. 1998). In this context, Botkin et al. (1998) define private information as “personal information over which individuals typically want and can exert control.” However, defining what information family members want to and are able to control is not an easy task. In the absence of consultation with family representatives, it may not be possible for investigators and institutional review boards (IRBs) to independently construct a “reasonable person” definition of privacy.

### Unanticipated or unwanted private information and confidentiality

An ethical challenge for environmental health research is that it may yield private information about medical conditions or genetic or environmental disorders or risks that are unanticipated, that may be distressing, or that parents may not want to know or not want their children to know. This raises issues of confidentiality: the duty to protect from disclosure of private information obtained by the investigator. This can be a problem for both the investigator and the family. For example, what should investigators do if they discover that a child in a normal control group has a biologic marker for an untreatable disease that typically emerges in early adulthood? What should families or the child be told if blood tests indicate that a 10-year-old research participant has been exposed to levels of an environmental toxicant associated with sterility? What if a misattributed paternity is discovered indicating that a child participant is not at risk for the health problem for which he or she was recruited into the study? Risks to self-interest may also emerge when asymptomatic children who have been informed about a genetic predisposition to environmental hazards must reveal in adulthood known health vulnerabilities on health insurance or employment applications [e.g., the case of an asymptomatic worker with a genetic susceptibility to exposure to beryllium barred by his company from continuing his position in a setting that exposed him to the agent (Marshall 1999)]. Such circumstances pose a dilemma between paternalistic policies that shield families or primary participants from information they may wish to know and irresponsible policies that place a burden on the family or child that cannot be remedied (Arnold et al. 1996; Wertz et al. 1994).

A risk of divulging unanticipated or unwanted private information obtained during the course of research is also economic, if health insurance is denied or more costly because research has identified a child as someone with a “preexisting” illness or at high risk for developing an environmentally based disorder (Arnold et al. 1996). The Canadian Paediatric Society (2003) “Guidelines for Genetic Testing of Healthy Children” advises against the genetic testing of healthy children for disorders that may arise in adulthood. The guidelines recommend restricting such testing to those conditions that arise in childhood. Based on the principle of respect for a child’s developing autonomy and the right to medical privacy, the guidelines reflect the view that each individual should have the right to decide when and if he or she wants to be tested for predisposition to adult-onset diseases and the right to control whether third parties will have access to this information. The Canadian Paediatric Society (2003) concludes that imposing on a child genetic testing for non-therapeutic purposes is unacceptable, even in the context of a family situation.

## Informed Consent

Informed consent is seen by many as the best means of protecting individual privacy rights in research. In both law and ethics, minors have been presumed to lack the capacity to provide informed consent because of immature cognitive skills, inadequate experiences in situations analogous to the research context, and the actual and perceived power differentials between adolescents, parents, and clinical researchers (Fisher 2002, 2003b; Grodin et al. 1994; IOM 2004). Under federal regulations, the privacy rights of minor children are protected by the requirement that informed guardian permission is obtained before a child can participate in research. The developing autonomy and privacy rights of children are further protected through regulations requiring that provisions are made for soliciting the assent of the child, when he or she is capable (DHHS 2005, 45CFR 46.408; U.S. EPA 2006, CFR 26.406).

### Children’s capacity to consent

Empirical studies suggest that children’s ability to understand the nature and purpose of research and their research rights begins to emerge in early childhood, reaching adult levels by mid-adolescence (Abramovitch et al. 1995; Bruzzese and Fisher 2003; Ruck et al. 1998a, 1998b). Research also suggests that irrespective of their more mature cognitive capacities, adolescents may not exert their research rights because of inexperience with health care or research decision making or out of fear of disapproval by parents, researchers, or clinical staff (Abramovitch et al. 1991; Broome 1999; Bruzzese and Fisher 2003; Scherer 1991; Susman et al. 1992). For example, interviews with children about their participation in genetic susceptibility research indicated that participants’ initial positive reaction to participation often reflected an inadequate appreciation of the risks and benefits of sharing test results with others or the uncertainties about testing (Bernhardt et al. 2003).

Despite cognitive and experiential immaturity, older children’s nonadult status and assent vulnerabilities do not justify ignoring their privacy rights. Rather, when feasible, environmental investigators can strive to create a goodness of fit between children’s maturing skills and the research context by approaching child assent as a process of research education fitted to the child’s age and abilities (Fisher 2002, 2003a). Given the evolving nature of analysis of stored data, the multivariate nature of environmental effects on children’s health, and privacy risks that may be unfamiliar or unanticipated at the onset of experimentation, an educational approach to environmental research can benefit parents as well as child participants. Developmentally fitted assent procedures can also help parents optimize children’s involvement in the participation decision and promote children’s nascent rights to privacy (Fisher 2003a).

## Cultural Attitudes toward Shared Information

Privacy decisions are also complicated by ethnic variation in attitudes regarding information sharing between parents and children (IOM 2005). For example, in some cultural groups, an investigator’s failure to provide information about a child’s health would be considered disrespectful of the parental role, whereas in other cultural groups disclosure of private information to family members is considered an intrusion on the parent–child relationship (Casas and Thompson 1991; Fisher 2002, 2003b; Fisher and Ragsdale 2006; Oetting and Beauvais 1990). Moreover, ethnic minority families, especially recently immigrated families, may be unfamiliar with local, state, and federal reporting laws regarding child abuse and neglect. In these contexts, typical blanket statements included in consent forms noting that confidentiality will be protected “except where reporting such information is required by law” may be uninformative at best and deceptive at worst (Fisher et al. 2002; Trimble and Fisher 2006).

Once language-appropriate and culturally valid criteria for collecting and disclosing private information have been established, whenever possible, investigators should identify culturally appropriate persons and agencies that can best serve the interests of families who may want a referral or immediate intervention for the participant risks identified by the research. By partnering with community members, schools, child welfare agencies, courts, law enforcement agencies, and health facilities, investigators can build culturally sensitive “systems of protection” that define when, how, and to whom environmental health data will be reported in a manner consistent with both participant cultural values and laws governing reporting (Fisher et al. 2002; IOM 2005; Minkler and Wallerstein 2003). According to the Centers for Disease Control and Prevention (CDC 1997), strategies to engage communities include capacity building, coalition building, and community organizing (see also Lerner et al. 2000).

## Studies of Genetic Predisposition to Environmentally Related Diseases

The Human Genome Project has paved the way to discovering individual differences in genetic predispositions to diseases associated with exposure to hazardous environmental materials. Findings from gene–environment epidemiologic studies have the potential to influence policies that reduce environmentally related diseases and create safer living and occupational environments. Information gained from research on the interactions between genes and environment may also be used by businesses to justify declining health insurance, housing, or employment. Responsible science therefore requires strict enforcement of confidentiality protections for individually identifiable information gained from such research.

### Sharing research-derived genetic information with parents

In situations in which investigators will share results of genetic testing with parents, the child has no say in whether others will be privy to one of the most private elements of individuality, one’s genetic makeup. In addition, a unique aspect of genetic research involving neonates or children is that others know highly personal information about them of which they are unaware and may remain unaware. Informing parents about a newborn or child’s genetic vulnerability to environmental disease discovered during research participation can be ethically appropriate when interventions exist that can reduce such vulnerability. In such cases, the principle of beneficence may have privileged status over the principle of respect for individual privacy. However, the same decision may not be morally appropriate in situations in which guardians are given information about their child’s genetic makeup when the chance of the child developing an environmental disease in the future is only probabilistic and treatment will not influence whether the child will develop the disease (Ross 2002).

Sharing with parents their child’s genetic susceptibility information obtained from asymptomatic children can unintentionally threaten the child’s best interest. Parents may treat children differently based on knowledge of their potential genetic predisposition to disease. Although parental efforts to avoid unnecessary environmental hazards may be a positive consequence of sharing children’s private genetic information, negative consequences can also occur. Parents may become over-protective or overly pessimistic about the child’s future, resulting in a restriction of activities and opportunities the child might have otherwise been afforded.

### Scientific documentation and available interventions

Decisions to share experimentally derived information about a child’s genetic makeup should rest on the certainty of scientific documentation. The weaker the scientific foundation is for a hypothesized relationship between a genetic test and an environmental disease, the weaker the moral argument to violate a child’s genetic privacy rights. The direct application of environmental health research to understanding causal mechanisms for environmental disorders of childhood can be limited. For example, when complex chemical exposures (e.g., air pollution) are investigated, biomarkers may reflect a reaction to one particular component of the mixture, when in fact the health disorder arises from another component (Soskolne 1997).

Disclosure of genetic information obtained through research should occur only if the hereditary nature of environmental disease susceptibility has been clearly demonstrated, the disease presents a major risk to the child’s future health, there is a low probability of false positives, and remedies are possible (Grandjean and Sorsa 1996). Along these lines, Annas et al. (1995) have argued that the right of parents to give permission to the collection and analysis of a child’s DNA be prohibited for children < 16 years of age for any condition that will not develop until adulthood, unless some effective measure can be taken before adulthood to prevent or ameliorate the disease. Such limitations on access to DNA information should be provided to the parents and child at the time of informed consent so that parents who do not agree with the restriction can refuse their child’s participation.

Research on genetic susceptibility to childhood asthma or adult lung cancer from early exposure to air pollutants provides a good example of this approach. Within this framework, it might be ethically permissible and, indeed, ethically responsible for investigators to inform parents about their child’s genotype if *a*) research had demonstrated a strong correlation between the genotype and development of environmentally induced asthma, *b*) the specific air pollutants triggering the asthmatic condition were identified, and *c*) it was possible to limit or remove the child from exposure to this pollutant. On the other hand, from a genetic privacy perspective, it might not be ethically responsible to inform parents about their child’s genotype if *a*) research indicated a low but significant correlation between the genotype and susceptibility to adult lung cancer, *b*) the specific element within a complex pollutant triggering environmental adult lung cancer had not been identified, and *c*) it was difficult to recommend with confidence an environment that would reduce any potential risk.

### The right not to know

Many adults do not wish to know their chances of developing an environmental disease. Federal guidelines permit the “right not to know” option in cases in which early treatment is not available [Office for Protection from Research Risks (OPRR) 1993]. Guidance is unclear concerning the role of guardians in determining a child or adolescent’s right to know or not know of a medical condition or environmental risk revealed through research. When children participate in genetic predisposition studies, they may be deprived of the opportunity afforded adult participants to refuse to be informed about their disease susceptibility. Thus, predictive genetic research involving children may violate both their right to refuse invasive data collection procedures and their right to withhold or not be told information that may be detrimental to their self-interests (Grandjean and Sorsa 1996). One approach to resolving this problem is to inform parents that children will be notified that such genetic information is available when the child turns 18 (Fisher et al. 1996)

### Distinctions between genetic testing for research versus treatment

The threshold for granting parents the right to give permission for the collection of their child’s genetic information without the child’s assent should be higher when such information is collected solely for research purposes than when it is collected as part of diagnostic assessment and treatment decisions that will directly affect the child’s health. Such a position is compatible with federal regulations permitting IRBs to approve research that allows parental permission to override child dissent when participation in a research study holds out a prospect of direct benefit to the child’s health or well-being available only in the context of the research (DHHS 2005, 45CFR46.408a).

## Prenatal Research and Maternal Privacy

Data collection during the prenatal period is important to ascertain aspects of the fetal environment that may contribute to congenital anomalies or later diseases related to maternal exposure to environmental toxicants. Agents investigated can include aspects of the physical environment of which the pregnant woman is unaware or that are unknown, such as air pollutants or lead contaminants, or agents the mother has intentionally ingested, such as antibiotics, antiallergens, hair dyes, illicit drugs, or high levels of alcohol for which prenatal risks are or are not known. Umbilical cord blood (UCB) is a frequently used source of information for identifying infant biologic markers and prenatal exposure to toxicants. Women are often asked to contribute UCB soon after delivery when they may not be fully informed about the type of personal data that will be stored in UCB banks (Sugarman et al. 1998). After consent, mothers may be asked about their sexual and medical history or their blood may be tested for infectious diseases, such as HIV and hepatitis. Prenatal studies may intrude on maternal privacy by asking whether pregnancies were conceived through reproductive technologies or provide information on a child’s paternity that was either unknown by the woman or that she did not wish to be revealed.

Private information about both the mother and fetus collected during the prenatal period may be stored in registries used in epidemiologic studies to monitor temporal or geographic patterns. To be of scientific and social value, registries must include minimal demographic information about the mother (i.e., age, ethnicity, and type of employment if it is relevant to potential exposure to environmental toxicants) and geographic region, as well as linkages between various sources of information about the mother and the fetus or newborn that at some point in the process must include a unique identifier that may pose privacy risks for the mother.

Investigators and IRBs need to be aware that risks to pregnant women outlined in this section raise issues of distributive social justice because the risks are assumed primarily by women rather than men. Thus, calculations of the ratio of scientific benefit to the confidentiality and privacy risks associated with such studies need to consider whether it is fair to burden one segment of the population with such risk.

### Maternal privacy risks during prenatal testing

Collection of maternal urine samples or amniotic fluid to identify toxicants that may be associated with congenital anomalies may uncover maternal use of illicit drugs or illegal use of prescription drugs that can lead to criminal investigation, a child welfare complaint, loss of food stamps, or loss of Supplemental Security Income. Identification of toxicants in biologic samples from the mother or infant may also lead to disqualifications regarding maternal employment opportunities, social stigma, legal risk, or self-recrimination (Harvey et al. 2002). For example, Grandjean and Sorsa (1996) describe an instance in which a manufacturing company excluded smokers from work sites with asbestos exposure because of data indicating smoking might increase susceptibility to asbestos-related lung cancer.

Another threat to privacy can arise when, for recruitment purposes, physicians are alerted to the need for research on a suspected teratogenic agent unique to a specific maternal demographic. In these circumstances, whether or not the mother agrees to participate in the study, her physician may ask questions or conduct invasive tests that would not otherwise be required, and her refusal to take such tests may stigmatize her as a neglectful or incompetent mother (Marshall et al. 2003). Additional privacy risks can emerge when women give permission for data from amniocentesis to be collected and the results of the amniocentesis lead to a decision to terminate the pregnancy—a decision that may be included in the data record.

### Maternal rights versus the information rights of other family members

Data on maternal exposure to or ingestion of teratogenic agents also raise questions regarding the rights of other family members to this information. Does the child’s biologic or legal father have the right to maternal environmental exposure information hypothesized to be linked to fetal or child health? Does the decision depend on the extent to which the hypothesized link has been empirically demonstrated? Although physicians may not be legally required to share such information, their participation in recruitment and data collection may be judged within a different set of ethical and legal medical principles.

Do adult children with congenital anomalies or other health problems associated with maternal exposures or ingestion of environmental toxicants have the right to know about their mother’s environmental history if the information is available in data banks? The answer to this question may rest on the source of information. For example, if information on the mother’s exposure or ingestion of the teratogen was acquired through analysis of amniotic fluid, fetal tissue, or UCB, one might conclude that the child, not the mother, was the data donor, with all the informational privileges attached to donor status. Ethical decisions regarding these complex issues must be made during the design phases of the research so that IRBs can help investigators ensure that mothers are adequately informed about the immediate or future risks to privacy they may face.

### Pregnant adolescents

Environmental health research involving teenage participants raise additional challenges in the arena of child and maternal rights. First, whether or not a pregnant teenager can provide legal independent consent to research participation depends on individual state laws governing the age and circumstances under which teenagers are considered mature or emancipated minors. In some states, for example, teenage mothers can provide consent for their infant’s participation in research but not for their own participation. Thus, in some instances, family members as legal guardians may have access to information about the adolescent mother’s exposure to or ingestion of teratogenic agents that would not be accessible if the mother were legally recognized as an adult (English 1995; Santelli et al. 1995).

A second concern arises in developmental studies of child and adolescent exposure to environmental toxicants. For such studies, adolescents may with parental permission assent to blood tests or other biologic assays to determine the presence of such toxicants. In some instances, these tests may indicate that the adolescent is pregnant, when the adolescent is not aware of the pregnancy and/or she does not want her parents to be notified. Disclosing such information to the adolescent may be perceived as a violation of privacy if the informed consent information did not include the possibility that a pregnancy could be detected. In other instances, the pregnancy may disqualify adolescents from continued participation in the study, leading to questions about the discontinuation from her parents. Is telling the parents the reason for discontinuation a violation of the adolescent’s confidentiality? Should investigators engage in deceptive explanations to parents about the reason for withdrawing the child from study participation? Should they continue to maintain the participant in the protocol with the intention of disqualifying the data, which in turn may violate their obligation to the agency funding the research? The best way to protect the privacy and confidentiality of female adolescent participants in these contexts is to plan in advance the disclosure procedures that will be most appropriate and least harmful to participants and their families and communicate what these procedures will be to both parents and adolescents during informed consent.

## Conclusion

Environmental health research has played and will continue to play a critical role in helping to identify environmental risks to children and to develop child-protective environmental policies. Research on the interaction between environmental agents and pediatric susceptibility has not kept pace with the large number of chemicals children are exposed to on a daily basis. Children’s physiology is different from that of adults and animals, and extrapolations from nonpediatric studies are in most cases insufficient to adequately determine pediatric environmental risk. Little remains known about genetic susceptibilities in children, and the vast majority of environmental agents have not been tested for pediatric toxicity. The need for environmental health research involving children has never been greater, nor has the need for consensus on how to conduct such research responsibly.

The moral claims of children in environmental health research are no different from those of adults. Children have the right to assume that scientists will communicate with them honestly, do them no harm, treat them fairly, and protect their autonomy and privacy. Respectful and compassionate research involving children requires understanding of children’s ways of thinking, their assent strengths and weaknesses, life experiences, and practical and family concerns. Environmental health investigators can help protect children’s privacy needs through developmentally fitted efforts to ensure that these claims are met (Fisher 2003a). For example, children have limited experience exercising their rights in response to requests from adult authority figures, especially within health care or other unfamiliar settings (Bruzzese and Fisher 2003). Constructing procedures that concretely demonstrate that dissent will not be penalized and providing opportunities to practice decision making can optimize voluntary participation choices. Additionally, the informed consent process for longitudinal studies and studies that will result in the creation of long-term databases must be viewed as a continuous process, with reconsent procedures that fit children’s maturing decisional capacities and parental concerns.
